# Neurological Consequences of Cardiac Arrhythmias: Relationship Between Stroke, Cognitive Decline, and Heart Rhythm Disorders

**DOI:** 10.7759/cureus.57159

**Published:** 2024-03-29

**Authors:** Swathi Srinivas, Bharath Vignesh RK, Venkat Nihar Ayinapudi, Aishwarya Govindarajan, Saran Sabapathy Sundaram, N Priyathersini

**Affiliations:** 1 Medicine, Sri Ramachandra Institute of Higher Education and Research, Chennai, IND; 2 Medicine, Chettinad Academy of Research and Education, Chennai, IND; 3 Pathology, Sri Ramachandra Medical College and Research Institute, Chennai, IND

**Keywords:** cardiac arrhythmia, stroke, dementia, cognitive deficit, atrial fibrillation (afib), brain degeneration, vascular neurology, interventional cardiology, interventional neurology, adult and geriatric clinical medicine

## Abstract

Cardiac arrhythmias are one of the most common disorders with high morbidity and mortality. The effect of cardiac arrhythmias on the brain is very pronounced due to the high sensitivity of the brain to oxygen and blood supply. This mortality is preventable by early diagnosis and treatment which improves the patient’s quality of life. Intervening at the right time, post arrhythmia is significant in preventing deaths and improving patient outcomes. Multiple pathophysiological mechanisms are studied for the brain-axis implications, that have the potential to be targeted by novel therapies. In this review, we describe the pathophysiological mechanisms and recent advances in detail to understand the functional aspects of the brain-heart axis and neurological implications post-stroke, caused by cardiac disorders.

This paper aims to discuss the current literature on the neurological consequences of cardiac arrhythmias and delve into a deeper understanding of the brain-heart axis, imbalances, and decline, with the aim of summarizing everything and all about the neurological consequences of cardiac arrhythmias.

## Introduction and background

Cardiac arrhythmia is a medical condition where the heartbeat has irregular rhythms, ranging from slow (> 60 beats/min) to fast (>100 beats/min), and can be present at any age. The most common cardiac arrhythmia is atrial fibrillation, which affects 33.5 million people worldwide, and it is well-documented as an independent risk factor for acute ischemic stroke. It is known that cardioembolic stroke is more severe and is linked to increased morbidity, and mortality. In addition, stroke patients with atrial fibrillation (AF) exhibit a high risk of recurrent ischemic events. It is crucial to detect AF for both primary and secondary stroke prevention [1,2]. Cardioembolic stroke is a frequent complication observed in the emergency department. It is believed that cardioembolism is the cause of one-quarter to one-third of all ischemic strokes. The referral population study found that the percentage of strokes thought to be cardiovascular increased from 22.88% in the 2002-2005 timeframe to 54.3% in the 2009-2012 timeframe, owing to the comprehensive cardiac workup carried out [1]. Cardio-embolic stroke can be caused by cardiac ischemia, while massive myocardial infarction (MI) can cause cardiovascular collapse. Cerebral hypo-perfusion can be linked to watershed-type infarction or diffuse cerebral hypoxic-ischemic encephalopathy. The rate of ischemic stroke in patients with MI within one month is 0.9%, and within a year after acute MI is 3.7%. Compared to those without complicated strokes, there is a significant increase in the one-year mortality rate [3]. 

This article aims to delve into the neurological manifestations seen in cardiac arrhythmias, its diagnosis, and current treatment protocols.

## Review

Cardiac arrhythmias: types and prevalence

Cardiac arrhythmias include a wide spectrum of rhythmic disorders caused by irregular electrical activity originating from the heart, thus disrupting the physiological synchronization of cardiac contractions. These disorders vary with symptoms, ranging from palpitations to life-threatening conditions.

The most common type identified globally is AF, which affects around 150-200 million people [4]. AF is identified by rapid and irregular electrical activity within the atria, which leads to an irregular heartbeat. AF has been identified as a significant risk factor for stroke and heart failure. Ventricular tachycardia (VT) and ventricular fibrillation (VF) present as rapid, but regular rhythms originating from the ventricles causing rapid ventricular activity, also identified as life-threatening conditions. Supra-ventricular tachycardia (SVT) includes a spectrum of arrhythmias that originate just above the ventricles and cause rapid heart rates but usually do not pose a threat to life [5]. Bradycardia, on the other hand, indicates a slow heart rate that can be physiologically normal in athletes, who experience no symptoms. While others present with lightheadedness, fainting, and weakness, with life-threatening symptoms with severe bradycardia.

The prevalence of arrhythmias strongly depends on various factors like age, underlying health conditions, and lifestyle. AF is the most prevalent and affects the older age group with a global prevalence of about 2-4% [6]. Ventricular arrhythmias like VT and VF are commonly seen in those with an underlying cardiac disease. SVT affects individuals of all age groups and may occur sporadically, without an underlying cardiac cause.

Hence, cardiac arrhythmias represent a diverse group of rhythm disorders, each with its distinguishing characteristics and prevalence patterns. While AF remains the most prevalent and increasing arrhythmia, ventricular arrhythmias pose higher life-threatening risks, especially in those with pre-existing cardiac conditions. Understanding the different arrhythmias and their prevalence is imperative for early identification and effective management, ultimately enhancing patient outcomes and reducing associated risks [7,8].

Mechanism and pathophysiology

The relationship between the heart and brain follows a very intimate path. Though physically discontinuous, the information is passed on via nerves, neurotransmitters, and ions. A circulatory change produced by a change in rhythm can have devastating effects on all tissues of the body, particularly the brain which is highly oxygen-sensitive. Any disruption in the blood supply to the brain can trigger various complications such as stroke, transient ischemic attack (TIA), cognitive dysfunctions, seizures, and dementia. Since stroke and dementia are the most common cerebral complications, this section will deal with the mechanism and pathophysiology of cardiac arrhythmias with dementia and stroke. Of all known rhythm imbalances, AF is the most common [9] and is known to produce a maximum decline in neurological function [10]. In the past, the association between AF and stroke was uncertain as often one was accused of causing the other. Subsequent studies reveal three explanations: 1) AF causes stroke, 2) stroke causes AF, and/or 3) AF is associated with other factors that cause stroke [11]. Aging and systemic vascular risk factors cause an abnormal atrial tissue substrate or atrial cardiopathy, that can result in AF and/or thromboembolism.

Despite various advances, the pathophysiology of AF remains unclear, most studies suggest a multifactorial process that results in the formation of AF. Data from several studies have demonstrated that in addition to preexisting cardiovascular diseases, obesity, hypertension, diabetes mellitus, and sleep-disordered breathing are associated with a higher risk of developing AF. The proposed pathophysiology suggests a “final common pathway” where these risk factors cause electrophysiological changes in atrial tissues. Alterations in the regulation of membrane channels and proteins result in abnormal electrical excitability. Atrial tissues, in particular pulmonary vein musculature, show a high degree of automaticity, resulting in ectopic beats (premature atrial contractions). Successive rapid atrial ectopics may then initiate either atrial tachycardia or frank AF. Additional cellular tissue remodeling results in abnormal conduction properties throughout the atria shortening of atrial tissue refractory periods. These functional and anatomic changes in atrial tissues appear to correlate with the progression of clinical AF. The data from various multi-centric studies indicate that “one of the most important consequences of AF is a significantly increased risk of stroke compared to the general population, causing ~25% of all strokes. The risk of dementia is increased in patients with AF, as is the risk of MRI-detected asymptomatic embolic infarct”. Once AF develops, the dysrhythmia causes contractile dysfunction and stasis, which further increases the risk of thromboembolism. In addition, over time the dysrhythmia causes structural remodeling of the atrium, thereby worsening atrial cardiopathy and increasing the risk of thromboembolism even further. As mentioned earlier, parallel to this is generalized risk factors such as age, hypertension, diabetes, smoking, and alcoholism alongside systemic risk factors like cardiovascular diseases, and underlying/preexisting vascular diseases which cause remodeling of the vascular tissue as an unavoidable outcome, which have the potential to trigger both these conditions such as large-artery atherosclerosis, ventricular systolic dysfunction, and in-situ cerebral small-vessel occlusion. Both these conditions follow a never-ending vicious cycle of Virchow’s triad [12].

**Figure 1:**
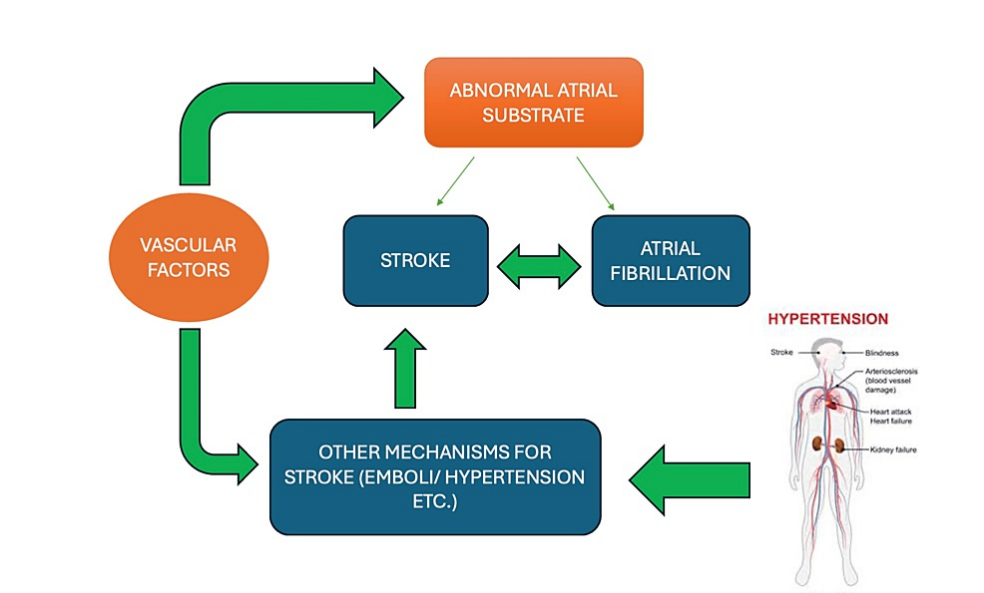
Mechanisms and pathophysiology The delicate interplay between cardiac arrhythmias, neurological deficits, and their sequelae [15] Image credit: Swathi Srinivas Flowchart drawn by author(s)

As shown in Figure 1, cardiac arrhythmias pose a threat to stroke which in turn is a cause for dementia [13]. Post-stroke cognitive impairment and dementia (PSCID) occur because of ischemic stroke caused by various factors including arrhythmias [14]. Although dementia and AF share several risk factors, there seems to be an independent history of clinical stroke and other comorbidities such as hypertension, heart failure, and diabetes. Proposed mechanisms linking AF to cognitive decline include altered hemodynamics resulting in cerebral hypo-perfusion, genetic factors, and silent cerebral ischemia due to subclinical microemboli, thereby posing a risk of stroke and dementia [15]. It has been proven that even a subtle but persistent drop in cardiac output can affect cerebral perfusion homeostasis and increase the risk of cognitive decline in the elderly [16,17].

The elevated incidence of stroke in AF patients explains the relationship between AF and the development of vascular dementia [18]. More recently, several studies have shown that AF is also linked with an elevated risk of other dementias, including Alzheimer’s disease, independent of clinical vascular events [19] Further, studies suggest that close monitoring of patients with AF and/or dementia is required to obtain accurate projections on the relationship between the two.

Neurological impact of cardiac arrhythmias

**Figure 2:**
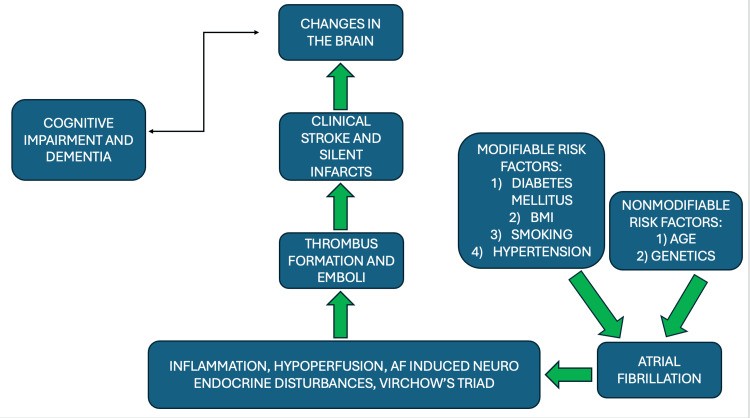
Neurological Implications of cardiac arrhythmias The risk factors and cause of changes in the brain due to cardiac arrhythmias [16] Image credit: Swathi Srinivas Figure drawn by author(s)

**Figure 3:**
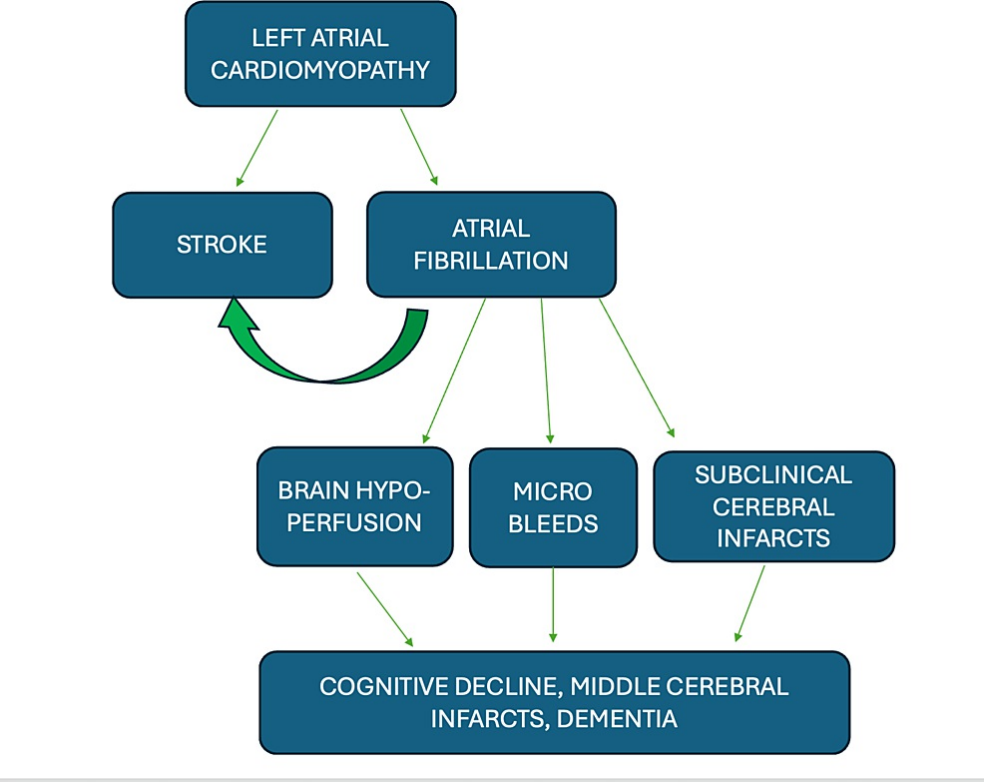
Sequence in cognitive decline post cardiac arrhythmia The flowchart illustrates the sequence of the onset of neurological deficits [17] Image credit: Swathi Srinivas Flowchart drawn by author(s)

Cardiac arrhythmias can have significant neurological associations due to their impact on blood flow, oxygen supply, and overall cardiac function (Figures 2, 3). Since the brain is highly contingent on constant and adequate blood flow, any disruption in the cardiac rhythm can affect cerebral perfusion, thus leading to neurological consequences.

Cardiac arrhythmias can lead to inadequate blood flow to the brain that can decrease cerebral perfusion thus leading to hypo-perfusion and ischemia. This in turn can cause neurological symptoms such as dizziness, syncope, and confusion [20]. Certain arrhythmias, especially AF can lead to the formation of blood clots in the atria. If these clots dislodge, they can form emboli, travel to the brain, and cause a stroke [21]. Arrhythmias may predispose patients to transient disruptions in blood flow to the brain resulting in brief episodes of neurological dysfunction producing transient ischemic attack [22]. Chronic or recurrent hypo-perfusion and ischemia may contribute to cognitive impairment over time. Conditions like vascular dementia can be exacerbated in the presence of cardiac arrhythmias. When there is a significant drop in cerebral perfusion, a cardiac arrhythmia may also trigger a seizure.

Further, cardiac arrhythmias can affect the autonomic nervous system which can lead to symptoms like palpitations, sweating, and nausea. These symptoms when prolonged can have a secondary impact on the nervous system. Severe or prolonged arrhythmias can lead to hypoxia. Since neurons are highly sensitive to oxygen levels, prolonged hypoxia can lead to neuronal damage and dysfunction of the nervous system [20].

Diagnostic approaches

The diagnostic approaches to assess the neurological consequences of cardiac arrhythmias involve a combination of clinical evaluation, imaging studies, and other investigations.

Primarily, a detailed clinical history and physical examination are done to elicit symptoms such as dizziness, syncope, cognitive changes, and other neurological manifestations. A thorough physical exam can be done to assess neurological function and deficits (if any). A standard 12 lead EKG can be used to detect and characterize cardiac arrhythmias. Further, a Holter monitor can be used to continuously monitor for cardiac arrhythmias, for as long as two months.

An ultrasound of the heart can assess its anatomical structure and physiological function, including the evaluation of cardiac valves, chamber size, and any structural abnormalities that may contribute to arrhythmias [23]. To assess blood flow to the carotid arteries as well as detect any emboli, a carotid ultrasound can be done [23]. Electrophysiological studies of the heart, although an invasive procedure, are very useful to map the electrical activity and identify the source of arrhythmia. It may also be useful therapeutically, in the case of catheter ablation [24].

Magnetic resonance imaging (MRI) of the brain with a diffusion-weighted imaging sequence can be used to assess the anatomical structure of the brain as well as to detect any abnormalities arising from ischemic events, strokes, or other neurological consequences [25]. Contrast computed tomography scan (C-CT scan) can detect vascular abnormalities in the brain, in practice it is used to detect acute neurological events such as hemorrhagic stroke [25]. Transcranial Doppler can be used to assess blood flow velocity in the cerebral and check for cerebral perfusion and potential emboli. Its use can be extended therapeutically to break down smaller emboli and thrombus dislodged in blood vessels [25].

Management and treatment

As mentioned earlier in mechanism and pathophysiology, Cardiac arrhythmias, characterized by abnormal heart rhythms, can disrupt blood flow and lead to the formation of blood clots. These clots then travel to the brain via arteries, causing ischemic strokes. In addition to this, arrhythmias result in decreased cardiac output, leading to cerebral hypo-perfusion and subsequent cognitive decline. Moreover, several studies have shown that AF, the most common type of cardiac arrhythmia, is associated with an increased risk of dementia and cognitive impairment. The management strategies devised are based on extensive studies and various proven treatment protocols, to combat the decline in neurological function and prevailing cardiac arrhythmias. The strategies deployed at present are listed as follows.

Detection of Arrhythmias and Monitoring Cardiac Function

A standard 12 lead ECG can detect an overt arrhythmia, while a paroxysmal arrhythmia with non-specific triggers, needs continuous monitoring for detection. Continuous ambulatory monitoring devices such as Holter monitors (24 hours to days-weeks) and implantable loop recorders to detect arrhythmias, are deployed in patients with risk factors for stroke and cognitive decline. Parallelly, clinicians should stress routine screening for cognitive function in patients with known cardiac arrhythmias to identify early signs of decline in neurological function.

Pharmacological Interventions

Anticoagulant therapy: Administer anticoagulants such as warfarin (vitamin K antagonist) or novel oral anticoagulants (NOACs) which are factor Xa inhibitors to reduce the risk of stroke in patients with AF. Advantages of NOAC include patients not requiring regular international normalized ratio (INR) monitoring as well as not having to consume a vitamin K-restricted diet. As the half-life of NOACs is lesser, dose reversal for undergoing procedures would be easier. However, to quantify the effect of anti-coagulation, changes in prothrombin time (PT), partial thromboplastin time (PTT), and INR levels are required. 

Supplemental usage with anti-platelets, clopidogrel, prasugrel, maybe indicated.

Rate and Rhythm Control

A custom pharmacological therapy based on the type and severity of the arrhythmia is required to optimize the heart rate and rhythm control, thereby reducing the risk of cerebral hypo-perfusion. This can be achieved with the usage of beta-blockers, calcium channel blockers, sodium channel blockers, and potassium channel blockers.

Interventional Procedures

Catheter ablation: Trans-catheter ablation for patients with refractory arrhythmias, particularly AF, to restore normal sinus rhythm and mitigate the risk of thromboembolic events. The ectopic zone is identified and ablated in one out of three ways, using thermal energy, radio frequency, or cryogenics, by doing so, the strip of cardiac muscle causing trouble is destroyed and henceforth the trigger would be nullified.

Device Therapy

Usage of cardiac devices such as pacemakers must be considered in case of severed left ventricular (LV) failure +/- ejection fraction, depending on the degree of failure a single chamber or a dual chamber pacemaker should be considered. Deploying a pacemaker not only fixes the arrhythmia but also acts as a barrier restricting neurological decline.

Lifestyle Modifications

Encourage adherence to a heart-healthy diet, regular exercise, and smoking cessation to reduce modifiable risk factors for both cardiac arrhythmias and neurological complications. Educate patients about the importance of medication adherence and regular follow-up appointments to optimize treatment outcomes and minimize the risk of disease progression. Providing affectionate tender loving care will boost the morale of the patient and increase their willpower to stick to treatment modalities.

Multidisciplinary Care

Collaborating with neurologists, cardiologists, geriatricians, and other healthcare professionals to provide comprehensive care for patients with cardiac arrhythmias and neurological decline is important in achieving a holistic successful outcome. Implement multidisciplinary care pathways and regular case conferences to facilitate communication and coordination among team members, ensuring holistic management of patient’s complex medical needs.

Research and future directions

Research on the neurological consequences of cardiac arrhythmias is an evolving field with ongoing investigations to have in-depth knowledge and to improve patient outcomes. Integrated cardiac neurological studies can bridge the gap between cardiology and neurology to understand the relationship between cardiac arrhythmias and their neurological consequences. Advanced imaging techniques such as functional MRI (fMRI) and diffusion tensor imaging (DTI) can be used to provide more detailed insights into structural and functional changes in the brain associated with cardiac arrhythmias. Further, Identification and validation of specific biomarkers can serve as indicators for neurological consequences in patients with cardiac arrhythmias, this can develop a more targeted approach for timely interventions.

Understanding the genetic and molecular mechanisms underlying both cardiac arrhythmias and their neurological consequences can help to develop a personalized intervention. The utilization of digital health and wearable technologies can help with the continuous monitoring of cardiac rhythms and neurological parameters. This can enhance early detection and intervention. Conducting research that focuses on the impact of cardiac arrhythmias on the quality of life and cognitive function can improve patient-centered care approaches. Designing and conducting clinical trials to test the efficacy and safety of novel therapeutic approaches for preventing or treating neurological consequences associated with cardiac arrhythmias. Finally, the application of artificial intelligence and its integration into machine learning algorithms for the analysis of large datasets including ECG and imaging data to identify patterns and predict neurological outcomes in patients with cardiac arrhythmias [26]. Focusing on these aspects can help guide clinicians in providing more personalized and effective care for individuals' outcomes. Collaboration between researchers and clinicians in this aspect is essential to advance the understanding of these complex interactions and develop innovative solutions for patient care.

## Conclusions

Both the heart and the brain follow a very intricate path and are very sensitive to hemodynamic changes. A cerebral insult may lead to a cardiac infarct, while a cardiac arrhythmia triggering thromboembolism results in a migrating thrombus, which occludes the cerebral vessels. Though the focal neurological deficit would be instantaneous, the consequences of it and the subsequent cognitive decline would be much more gradual in onset. The nature of neuro-functional decline is dependent on patient risk factors and the strong genetic connection associated with it. Despite the availability of various investigations and treatment options, the diagnostic method of choice is case-specific, and doctor-specific. A uniform treatment regime is hard to set up as the extent of the disease is case-specific. It is also noteworthy to observe that a decline in mental health is commonly noted among patients who have been receiving long-term treatment. Clinicians should keep in mind the status of their patient’s mental health and focus on a holistic recovery.

In conclusion, it is necessary for treating physicians to look out for cognitive decline in patients with cardiac arrhythmias and investigate at the right time to provide the most appropriate intervention for the patient to improve the general outcome and provide a better quality of life to the patient.
